# The *ezh2(sa1199)* mutant zebrafish display no distinct phenotype

**DOI:** 10.1371/journal.pone.0210217

**Published:** 2019-01-24

**Authors:** Bilge San, Julien Rougeot, Kai Voeltzke, Gertie van Vegchel, Marco Aben, Karolina M. Andralojc, Gert Flik, Leonie M. Kamminga

**Affiliations:** 1 Radboud University Medical Center, Radboud Institute for Molecular Life Sciences, Nijmegen, The Netherlands; 2 Radboud University, Faculty of Science, Department of Molecular Biology, Radboud Institute for Molecular Life Sciences, Nijmegen, The Netherlands; 3 Department of Animal Ecology and Physiology, Radboud University, Nijmegen, The Netherlands; Imperial College London, UNITED KINGDOM

## Abstract

Polycomb group (PcG) proteins are essential regulators of epigenetic gene silencing and development. The PcG protein enhancer of zeste homolog 2 (Ezh2) is a key component of the Polycomb Repressive Complex 2 and is responsible for placing the histone H3 lysine 27 trimethylation (H3K27me3) repressive mark on the genome through its methyltransferase domain. Ezh2 is highly conserved in vertebrates. We studied the role of *ezh2* during development of zebrafish with the use of a mutant allele (*ezh2(sa1199)*, R18STOP), which has a stop mutation in the second exon of the *ezh2* gene. Two versions of the same line were used during this study. The first and original version of zygotic *ezh2(sa1199)* mutants unexpectedly retained *ezh2* expression in brain, gut, branchial arches, and eyes at 3 days post-fertilization (dpf), as revealed by in-situ hybridization. Moreover, the expression pattern in homozygous mutants was identical to that of wild types, indicating that mutant *ezh2* mRNA is not subject to nonsense mediated decay (NMD) as predicted. Both wild type and *ezh2* mutant embryos presented edemas at 2 and 3 dpf. The line was renewed by selective breeding to counter select the non-specific phenotypes and survival was assessed. In contrast to earlier studies on *ezh2* mutant zebrafish, *ezh2(sa1199)* mutants survived until adulthood. Interestingly, the *ezh2* mRNA and Ezh2 protein were present during adulthood (70 dpf) in both wild type and *ezh2(sa1199)* mutant zebrafish. We conclude that the *ezh2(sa1199)* allele does not exhibit an *ezh2* loss-of-function phenotype.

## Introduction

In biology in general and certainly in the field of zebrafish biology, there is a major interest to understand how individual proteins contribute to development and tissue homeostasis. The primary approach to achieve this goal is generating loss-of-function mutations in protein coding genes. Specific and inducible genome editing through site-directed mutagenesis by zinc finger nucleases, TALENs, or CRISPR-Cas9 [1–3], are routine now, but these approaches are still expensive and time consuming. Knockdown by morpholino treatment, on the other hand, is a relatively quick assay to produce phenotypes; however, this approach requires rigorous controls [4]. As much as 18% of morpholinos has been predicted to have off-target effects [5], and in some cases morpholinos lead to p53-activation-related cell death [6]. Moreover, morpholino effects persist no longer than about 5 days post-fertilization (dpf), and this makes studies on larval stages beyond 5 dpf virtually impossible. For reverse genetic screens, random mutagenesis is favorable for efficiency and cost-effectiveness. Alkylating agents such as ENU (N-ethyl-N-nitrosourea) and EMS (Ethyl methanesulfonate) have been used extensively in the last decades due to their high in-vivo potency for random mutagenesis. The development of the TILLING (Targeting Induced Local Lesions in Genomes) method at the start of the 2000s [7] has revolutionized zebrafish functional genomics [8] by enabling larger and faster forward and reverse genetics screens. The development of this method resulted in the Zebrafish Mutation Project in the Wellcome Trust Sanger Institute [9], with extensive collaborative effort. The project aimed to produce a complete set of mutant zebrafish lines for every protein-coding gene. F3-generation embryos, spawned by heterozygous F2 generation in-crosses, were monitored until 5 dpf for phenotypes. Mutants and carrier alleles from the Zebrafish Mutation Project are publicly available [9].

Mutant alleles serve as a great tool to study (the impairment of) gene regulation during embryonic development. Although every cell in an organism has the same DNA sequence, cells (and tissues) gain different functions during development through regulation of gene expression. Epigenetics is the field of biology which explains how covalent modifications on the genome affect gene expression, and as a result, cellular function. Research on the function of proteins involved in the epigenetic control of gene expression takes a crucial role in our understanding of how correct development and tissue homeostasis proceed. The Polycomb Group (PcG) proteins are essential transcriptional gene repressors which are highly conserved in all vertebrates. PcG proteins function mainly in two Polycomb Repressive Complexes (PRC): PRC1 and PRC2. PRC2 places the repressive histone H3 lysine 27 trimethylation (H3K27me3) mark on the genome through the methyltransferase Ezh2. This mark recruits PRC1, which is thought to stabilize the H3K27me3 mark through the mono-ubiquitylation of histone H2A lysine 119 (H2AK119Ub1) by the RING family of E3 ubiquitin ligases. These two interrelated functions of PRC1 and PRC2, in turn, cause compaction of DNA around histone proteins, and are thought to impede transcription by limiting the RNA Polymerase II accessibility to specific genes [10–12]. PcG mutations have received particular attention due to their association with embryonic lethality and cancer in placentals [13]; therefore, correct characterization of PcG protein function is essential for treatment prospects in human diseases [14,15].

Within PRC2, the methyltransferase Ezh2 has been extensively studied for its catalytic activity. The Ezh2 protein has two domains, WD-binding (the WD40-repeat-containing domain) and SET (the Su(var)3-9, Enhancer-of-zeste and Trithorax), which are also highly conserved in vertebrates [16]. The WD-binding domain, located near the N-terminus (amino acids 39–68 in zebrafish [16]) predominantly regulates the interaction between PRC2 components Eed and Ezh2 [17,18]. The SET domain, located near the C-terminus (amino acids 626–747 in zebrafish [16]) regulates the methyltransferase function of the Ezh2-PRC2 complex [19]. In mice, Ezh2 knockout causes early embryonic lethality [20], therefore, its function has been investigated through conditional tissue-specific knockouts [21–28]. According to the majority of these studies, the functions of Ezh2 in mice can be summarized in three categories: Ezh2 is crucial for normal cell proliferation in tissues, it regulates correct transcription of tissue specific genes, and it contributes to correct tissue maintenance. For instance, Ezh2-overexpressing hematopoietic stem cells that are transplanted into immune-compromised mice restore long-term (blood) repopulation potential; stem cell exhaustion is thereby prevented [29]. Recently, there is accumulating evidence for redundancy of Ezh1 in setting the H3K27me3 mark [30], albeit with lower activity [31], that may prevent the full PRC2-null phenotype as a result of tissue specific Ezh2 knockout. Tissue specific knockout of Eed, another essential subunit of the PRC2 complex, on the other hand, prevents formation of the PRC2 complex and results in complete loss of the H3K27me3 mark [32].

Early zebrafish development is highly synchronized under laboratory conditions, rendering this model organism suitable for epigenetic research. Moreover, the possibility to obtain 200–600 embryos at the same stage of development (per clutch) facilitates the sampling process for experimental techniques in epigenetics, which require large numbers of cells/donors. Studies on the establishment of epigenetic marks during early zebrafish development have given important insights on how the repressive functions of PcG proteins instruct developmental programs before tissue specification [33–35]. However, how the PRC2 complex regulates zebrafish organogenesis and tissue homeostasis is not completely understood. Therefore, studying PcG function in (mutant) zebrafish after tissue specification can shed light on epigenetic regulation of tissue homeostasis.

We previously studied the loss of *ezh2* during early zebrafish development with the mutant allele *ezh2(hu5670)*, which presents a nonsense mutation upstream of the methyltransferase domain [16]. Accordingly, maternal-zygotic *ezh2(hu5670)* mutant embryos form a normal body plan despite major differences in gene expression during the early hours of development compared to wild type embryos and die at 2 dpf exhibiting a range of phenotypes, including loss of myocardial integrity and suspected terminal differentiation defects in liver and pancreas. Zygotic *ezh2(hu5670)* mutants show no apparent phenotype at 5 dpf, but die around 11 dpf with intestinal and hepatic maintenance defects [36]. Similarly, zygotic *ezh2(ul2)* nonsense mutants generated by Dupret and colleagues [37] with the use of TALENs technology [3] show intestinal and pancreatic maintenance defects and lethality at 12 dpf. Moreover, these *ezh2(hu5670)* and *ezh2(ul2)* mutants present reduced H3K27me3 levels. Overall, these key studies support an essential role for Ezh2 in the epigenetic control of tissue maintenance and survival in zebrafish.

The study described here, aimed to investigate the effect of the loss of zygotic *ezh2* expression during zebrafish development. In an attempt to further our insight on Ezh2 function, the mutant allele *ezh2(sa1199)* generated by the Zebrafish Mutation Project was studied. From our observations on the development and survival of *ezh2(sa1199)* embryos, the original line provided had background mutations, displaying lethality around 2 dpf and a non-specific edema phenotype in mutants and wild types alike. We subsequently obtained another *ezh2(sa1199)* line in which background mutations were eliminated by selective breeding. Unexpectedly, in these mutants, no embryonic or larval phenotypes were observed, and the fish survived until adulthood. We conclude that the *ezh2(sa1199)* nonsense mutant line does not present a loss-of-function phenotype.

## Materials and methods

### Zebrafish husbandry and strains

All adult fish were maintained under standard conditions in a 14:10 light/dark cycle. Embryos were reared in E3 medium at 28.5°C [38] and staged following Kimmel *et al*. [39]. Two different versions of the *ezh2(sa1199)* nonsense mutant allele (R18STOP, CTGGAGGCGG**C>T**GAGTGAAGTC) were obtained from the Zebrafish Mutation Project [9], through Zebrafish International Resource Center. Mutant embryos were generated by in-crossing heterozygous carriers. Adult lines were renewed and maintained by out-crossing the carrier line with wild type Tubingen Long Fin (TLF) adults. European animal welfare laws and protocols were strictly followed and approved after ethical testing by Central Committee for Animal Experimentation (CCD) of the Netherlands in all experimental procedures.

### Genotyping

Embryos (2–3 dpf) were briefly anesthetized in 2-phenoxyethanol (0.1% v/v) and their caudal fins were clipped with a clean microsurgical blade. Individual fin-clips were lysed in 25 mM NaOH and 0.2 mM EDTA at 95°C and subsequently neutralized with an equal volume of 40 mM Tris-HCl (pH predicted to be ~5). Three different genotyping methods were developed. For amplification prior to Sanger sequencing, forward (5’-CATGGACATCTTTGGGTCCT-3’) and reverse (5’-ACACACATGCAACTGGACTC-3’) primers were used. Allele specific genotyping was done by combining a common forward primer (5’-AGATGTGCACTCCTACGTTTGATAC-3’) with reverse primers that differentially detect mutant (5’-GCATGTACTCAGACTTCACT**A**AC-3’) and wild type (5’-GCATGTACTCAGACTTCACT**A**GC-3’) alleles, respectively, allowing 1 mismatch (in bold). The mutation site is underlined. The third method applied a PCR amplification (forward 5’-ATGGGATTGACCGGGAGGAAATC-3’, reverse 5’-CTCTCTGGTTCCACGCAAGGAG-3’) followed by enzymatic digestion with HphI; due to the C>T mutation, the mutant allele gains a HphI restriction site, while the PCR product of the wild type allele remains uncut upon HphI incubation. All primers flanked the mutation site in exon 2.

### Whole mount in-situ hybridization

Embryos (3 dpf) were fixed in 4% paraformaldehyde in PBS (w/v) overnight at 4°C, and whole mount in-situ hybridization was performed as previously described [16]. The stained embryos were mounted in 3% methylcellulose in water (w/v). The mounted embryos were covered with a thin layer of PBST during stereo-microscopic (Leica MZ FLIII) imaging.

### Survival assay

Embryos (3 dpf) were genotyped by a combination of PCR and HphI digestion as described above. Wild-type (N = 18) and mutant (N = 21) larvae were transferred to the juvenile husbandry system in separate tanks at 5 dpf. Dead larvae were collected daily to assess survival.

### Quantitative real time PCR

Wild type (N = 2) and *ezh2(sa1199)* mutant (N = 2) adult siblings at 70 dpf were mechanically homogenized in TRIzol (Thermo Fisher) and total RNA was isolated as described elsewhere [40]. Total RNA was reverse transcribed with Superscript III (Invitrogen, 18080093) and poly-dT primers. Standard qPCR was performed with the use of SYBR Green (iQ SYBR Green Supermix, BioRad, 1708880) to detect mRNA levels of *ezh1* and *ezh2*, relative to reference genes *β-actin* and *ef1α* in technical triplicates. RT-qPCR primers are shown in S1 Table.

### Western blotting

Adult (70 dpf) wild type and mutant siblings of the *ezh2(sa1199)* allele were sacrificed in 2-phenoxyethanol (0.2% v/v). Individual fish were decapitated and the body was lysed in protein lysis buffer (50mM Tris pH 7.5, 150mM NaCl, 1% NP-40, 0.1% sodium deoxycholate, Roche protease inhibitor cocktail). After homogenization, 30 μg protein sample was loaded on a 12% SDS-PAGE gel. Fixed larval (7 dpf) wild type and mutant siblings of the *ezh2(sa1199)* allele were lysed in 50 μl RIPA buffer (20 mM Tris-HCl pH 8.8, 150 mM NaCl, 1% NP-40, 0.5% Sodium deoxycholate) in pools of 5, and SDS was added to reach a final concentration of 2% (v/v). Protein samples were incubated in a thermomixer at 600 rpm for 20 minutes at 100°C, followed by 2 hours at 80°C. Samples were subsequently sonicated 2 times for 15 seconds (Pico Bioruptor), cleared by centrifugation at 4°C at 16,000 g for 12 minutes, and loaded on a 4–15% gradient protein gel (Biorad, Mini-Protean TGX). Adult (70 dpf) and larval (7 dpf) SDS-PAGE bands were transferred onto nitrocellulose membranes for anti-Ezh2 (Cell Signaling Technologies, Ezh2 (Cell Signaling Technology, D2C9 XP), anti-β-actin (Sigma), and anti-H3K27me3 (Millipore) antibody staining. After incubation with the secondary antibody (Life Technologies, Alexa Fluor 800), the signal was visualized via Odyssey CLx Western Blot Detection System, and the anti-H3K27me3 bands were quantified by the gel analysis tool on Image J software, relative to anti-β-actin loading control.

### Assessment of water quality

System water samples (12–15 ml) from erroneous and correct piping were collected and acidified with 1–5% (v/v) HNO_3_ to keep metals in solution. Aluminum (Al), Calcium (Ca), Cadmium (Cd), Copper (Cu), Iron (Fe), Potassium (K), Magnesium (Mg), Manganese (Mn), Sodium (Na), Phosphorus (P), Sulfur (S), Silicium (Si), and Zinc (Zn) absorptions were measured by inductively coupled plasma—optical emission spectrometry (ICP-OES). Concentrations were calculated in parts per billion (ppb) and a ratio of erroneous and correct piping was taken for comparison of element concentrations.

## Results

**Fig 1:**
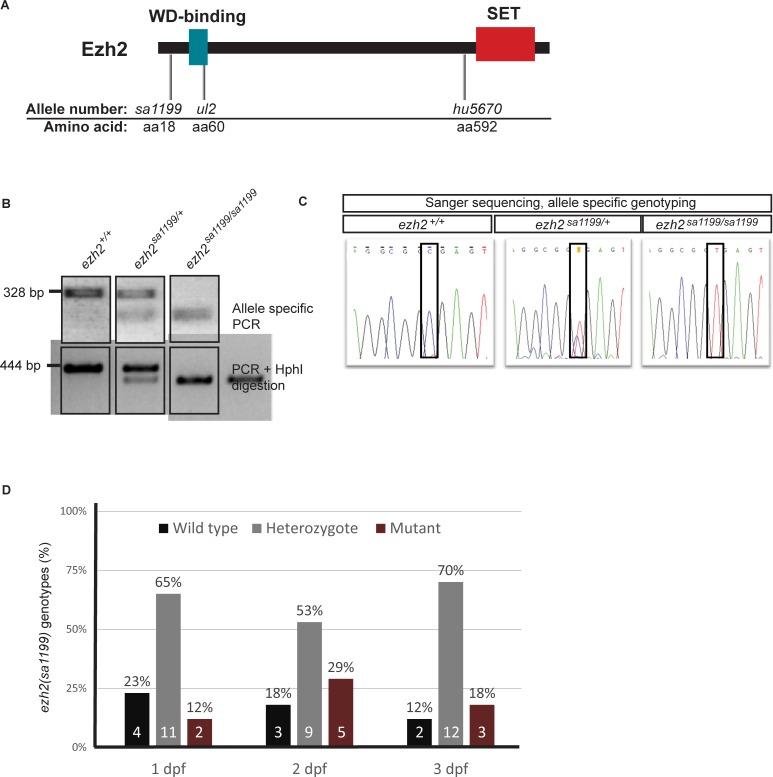
Validation of *ezh2(sa1199)* genotypes. **A.** Ezh2, its domains, and mutant allele positions (grey lines). The green and red boxes indicate WD-binding and SET domains, respectively. The *ezh2(sa1199)* allele (left) has a stop mutation on the arginine 18 position (R18STOP), *ezh2(ul2)* allele (middle) has a 22 bp insertion that leads to a nonsense codon at amino acid 60, and *ezh2(hu5670)* allele (right) has a nonsense mutation on the arginine 592 (R592STOP) position. **B.** Allele specific (top) and PCR-restriction (bottom) genotyping on caudal fin clips of 2 dpf embryos. Agarose gel electrophoresis shows differential amplification or restriction enzyme digestion of the alleles. A wild type, heterozygous, and mutant genotype from a single experiment is represented for each method. **C.** Allele-specific genotyping validation by Sanger sequencing. Two dpf embryo fin clips were allele-specifically genotyped and Sanger-sequenced to validate the genotyping method. The mutation locus (black box) is visualized for wild type (left), heterozygous (middle), and mutant (right) embryos. **D.** Allele specific genotyping of 1, 2, and 3 dpf *ezh2(sa1199)* in-crossed embryos (N = 17 per day). The percentage of wild types (black), heterozygotes (grey), and mutants (brown) show a Mendelian ratio. The number of embryos is indicated inside the bars of the graph in white.

To study the loss of *ezh2* during zebrafish development, we scanned the Zebrafish Mutation Project [9] for available loss-of-function mutations for this gene. We identified the *ezh2(sa1199)* allele, which has a nonsense mutation that causes a truncation at arginine 18 (R18STOP) in exon 2. Nonsense mutations in the same gene are predicted to show allelic heterogeneity, *i*.*e*. displaying similar phenotypes. This third mutant allele could complement the published mutant alleles *ezh2(ul2)* [36] and *ezh2(hu5670)* [16], which have nonsense mutations at amino acids 60 and 592 (Fig 1A), respectively.

### Genotyping *ezh2(sa1199)* mutants

Next to Sanger sequencing, we developed two genotyping methods to distinguish wild type, heterozygous, and homozygous mutant siblings. The *ezh2* mutant embryos were generated by in-crossing *ezh2(sa1199)* heterozygous adults. The two methods, allele-specific PCR amplification of the region flanking the mutation site and PCR amplification combined with restriction digestion by the enzyme HphI, both confirmed the predicted genotypes (Fig 1B). Allele specific PCR genotyping was verified by Sanger sequencing (Fig 1C). By all methods, *ezh2(sa1199)* sibling genotypes were confirmed to be in line with the predicted Mendelian ratio after an in-cross (Fig 1D).

### The *ezh2(sa1199)* zebrafish line expresses *ezh2* and shows a non-specific phenotype

**Fig 2:**
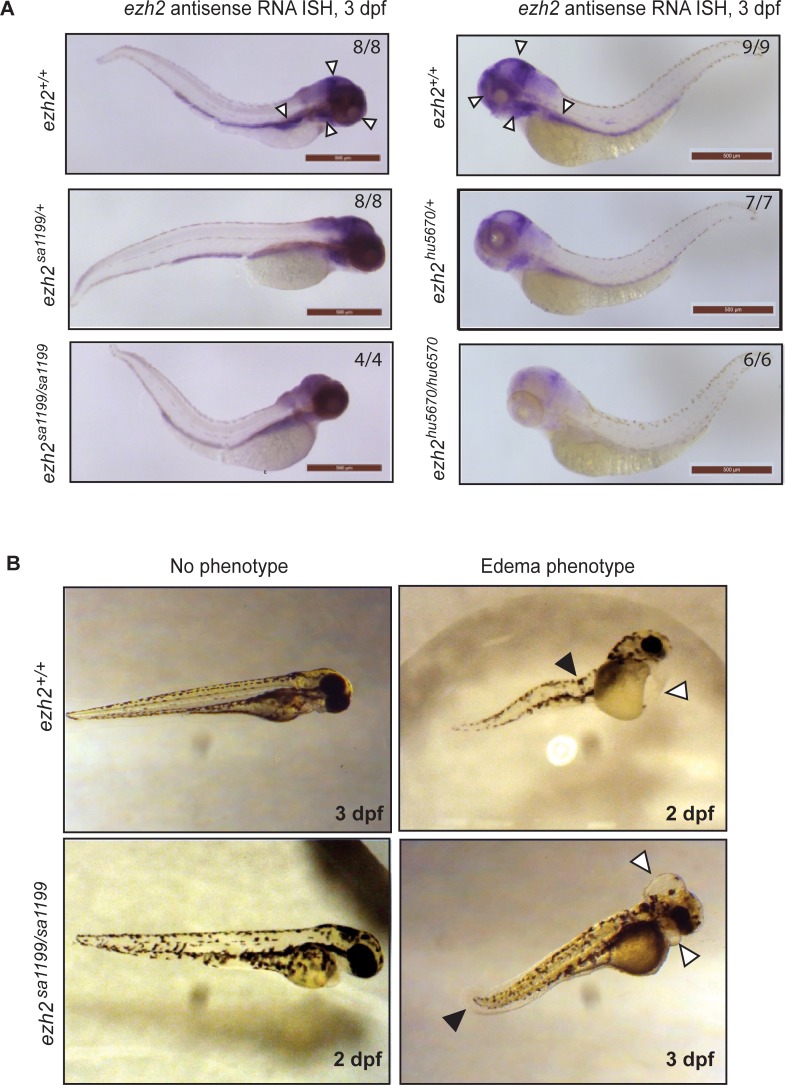
*ezh2(sa1199)* zebrafish line has non-specific phenotypes. **A.** Whole mount in-situ hybridization for *ezh2* on 3 dpf *ezh2(sa1199)* (left panel) and *ezh2(hu5670)* (right panel) embryos. *Left panel*. Wild type siblings (top) show *ezh2* expression in the brain, eyes, branchial arches, and the gut (white arrowheads). Heterozygotes (middle) and *ezh2(sa1199)* mutants (bottom) show the same expression pattern; they are phenotypically comparable to wild types and have the same spatiotemporal *ezh2* expression pattern. *Right panel*. Wild type siblings (top) show similar *ezh2* expression to *ezh2(sa1199)* in-cross embryos (white arrowheads). Heterozygotes (middle) show decreased expression, and *ezh2* is barely detectable in *ezh2*(*hu5670*) mutants (bottom). Scale bar: 500 μm. Numbers indicate the number of embryos displaying the shown expression pattern per total number of embryos tested. **B.** Left panel shows normal wild type and mutant embryos at 3 and 2 dpf, respectively. The right panel shows 2 and 3 dpf wild type and mutant embryos, respectively, with yolk sac, heart, and brain edemas (white arrowheads) and spinal curvatures (black arrowheads).

In mutant alleles such as *ezh2(sa1199)*, which translate into a stop codon, nonsense mediated decay (NMD) mechanisms are predicted to operate at the mRNA level [41–43]. Therefore, we predicted little to no *ezh2* expression in *ezh2(sa1199)* mutant embryos by 3 dpf. To test this, we performed whole mount in-situ hybridization (WISH) for expression of *ezh2* mRNA in *ezh2(sa1199)* mutant embryos. Surprisingly, *ezh2* mRNA expression patterns were highly comparable between wild types, heterozygotes, and homozygous mutant embryos at 3 dpf (Fig 2A, left panel). In all embryos tested, *ezh2* expression was visible in brain, eyes, branchial arches, and gut. This observation contrasts strongly with earlier studies [16,36,37], where *ezh2* expression in mutant embryos is severely suppressed at 3 dpf. Indeed, WISH on zygotic *ezh2(hu5670)* in-crossed siblings showed that *ezh2* expression is decreased in heterozygotes and about undetectable in mutants by 3 dpf (Fig 2A, right panel).

During the development of *ezh2(sa1199)* siblings, severe abnormalities were seen, both in homozygous mutant and in wild type embryos. Brain and heart edemas were present, with defects in blood circulation and spinal curvature throughout clutches of embryos from different parents (Fig 2B). This phenotype affected survival of the embryos. Although increased lethality was observed amongst mutants as of 2 dpf, embryos from all genotypes showed decreased survival. Swim bladder development was also affected, which might have contributed to lethality in larvae after 5 dpf. Zebrafish larvae which cannot inflate their swim bladders cannot rise to the water surface to reach good quality feed, and eventually die during early larval stages.

One may predict that lines derived from ENU-mutagenized libraries will retain background mutations. The effect of these possible mutations on embryonic phenotypes can be circumvented by selectively breeding the adult carrier pairs which produce healthy embryos, and subsequently out-crossing them with wild types. Such an *ezh2(sa1199)* line was obtained from the Wellcome Trust Sanger Institute.

### Out-crossed *ezh2(sa1199)* mutants show no lethality

**Fig 3:**
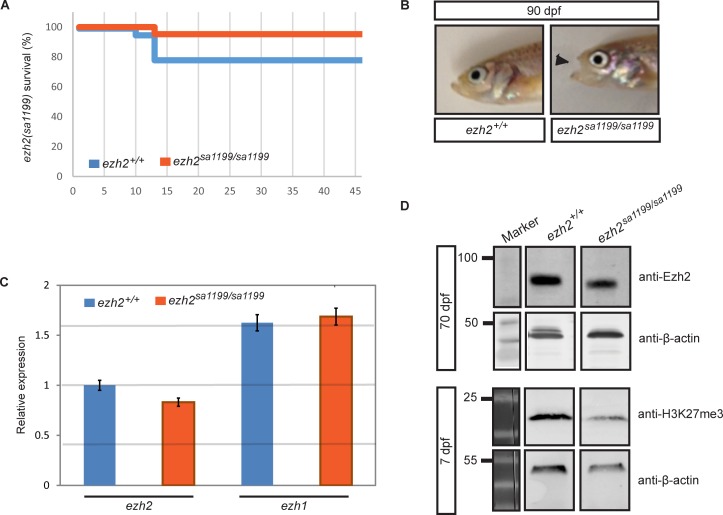
Selectively out-crossed *ezh2(sa1199)* survives until adulthood. **A.** Survival graph of *ezh2(sa1199)*. Survival assay was performed with *ezh2(sa1199)* in-crossed wild type (N = 18, blue line) and mutant (N = 21, orange line) siblings until 46 dpf. Some larval lethality during raising was seen both in wild types and mutants. The graph indicates that the survival of homozygous *ezh2(sa1199)* mutants is not significantly different from wild type siblings. **B**. Pictures of wild type and mutant *ezh2(sa1199)* adult siblings. While wild types have normal jaws (left), adult *ezh2(sa1199)* mutants (right) display an open mouth phenotype (black arrowhead). **C.** RT-qPCR measurement of *ezh1* and *ezh2* mRNA in *ezh2(sa1199)* adults. The (decapitated) body of 70 dpf wild type (blue) and mutant (orange) individuals were lysed and the presence of *ezh1* and *ezh2* mRNA was quantified and normalized to the reference genes *ß-actin* and *ef1a*. The *ezh1* (p = 0.9509) and *ezh2* (p = 0.1493) mRNA levels were not significantly altered in mutants. **D.** Ezh2 protein in single 70 dpf adults and H3K27me3 in 7 dpf pooled larvae. The presence of Ezh2 was tested in wild type (N = 3, left) and mutant (N = 4, right) bodies at 70 dpf, where Ezh2 expression persists (upper panel). At 7 dpf, mutant larvae (right) show 41.8% decreased levels of the H3K27me3 mark compared to wild type (left) larvae (lower panel) and relative to β-actin loading control. For each Western blot, representative bands were cropped from the same blot and shown in boxes for clarity of presentation.

During the development of out-crossed *ezh2(sa1199)* embryos, no non-specific phenotypes or edemas were observed. Next, survival of the out-crossed line was assessed by comparing the viability of wild type and mutant larvae. Surprisingly, no lethality in homozygous mutants occurred, as the fish successfully reached adulthood (Fig 3A). This observation contrasts strongly with recent data on zygotic *ezh2*(*sa1199*), *ezh2(hu5670)*, and *ezh2(ul2)* mutants, which survive on average for 7, 11, and 12 days, respectively [44,36,37].

By the time the *ezh2(sa1199)* line reached adulthood, all observed mutants developed an abnormal jaw structure; the mutant fish could not close their mouth, while jaws of wild type siblings were normal (Fig 3B). Concurrently, during a routine water quality check for various elements, a higher (33.46 ppb) than normal (1 ppb) copper concentration was measured in the system where wild type and mutant zebrafish in the *ezh2(sa1199)* background were housed (S1A Fig). High copper concentrations are known to cause developmental defects and mortality, particularly due to cellular stress of ion transporting cells in the gill epithelium in zebrafish [45] and tilapia [46]. Gills develop from the pharyngeal arches which house craniofacial cell precursors [47]. Initially, because the open mouth phenotype was detected only in the mutants, we suspected a delayed Polycomb group protein-related phenotype. Mice with conditional *Ezh2* knockout [48] and zebrafish mutant for *rnf2*, the catalytic subunit of PRC1 [49], do have craniofacial abnormalities. However, whether the craniofacial phenotype seen in homozygous mutants is related to *ezh2* loss is inconclusive.

Existing literature and earlier observations in our laboratory strongly favor the notion that *ezh2* mutant zebrafish have a Polycomb group protein-related phenotype and do not survive until adulthood. Yet, the observation that *ezh2* mRNA may be exempt from nonsense mediated decay in *ezh2(sa1199)* mutants (Fig 2A, left panel) prompted the assessment of Ezh2 mRNA and protein levels in 70 dpf wild type and *ezh2* mutant adult zebrafish. We euthanized and decapitated *ezh2(sa1199)* mutant and wild type individuals and lysed the body for real-time quantitative PCR analysis of *ezh1* and *ezh2* mRNA (Fig 3C) and Western blot analysis of Ezh2 protein (Fig 3D, upper panel, S1B Fig). No significant differences were detected in *ezh1* and *ezh2* mRNA levels between *ezh2(sa1199)* mutants and wild types. Ezh2 protein was detectable in the body in all samples, regardless of genotype (Fig 3D, upper panel), whereas it was absent in the head of wild types and mutants (S1B Fig). Interestingly, at 7 dpf, levels of the repressive H3K27me3 mark were decreased 41.8% in *ezh2(sa1199)* mutant larvae compared to wild types (Fig 3D, lower panel, S1C Fig). The anti-Ezh2 antibody utilized for this experiment is known to be specific for zebrafish and is absent in maternal-zygotic *ezh2* mutants (S1D Fig) [16,50]. Thus, in *ezh2(sa1199)* mutants, *ezh2* mRNA is not subjected to NMD and despite the presence of an R18STOP mutation, Ezh2 protein is still translated.

## Discussion

This study aimed to investigate the effects of zygotic loss of *ezh2* during zebrafish development. Our hypothesis was that zygotic *ezh2* mutant zebrafish would develop defects in tissue maintenance and consequently die at early larval stages. This hypothesis was supported by our earlier published research on zygotic *ezh2(hu5670)* mutants [36] and by Dupret and colleagues on the *ezh2(ul2)* allele [37]. In zygotic *ezh2(hu5670)* and *ezh2(ul2)* mutants, larval intestines are underdeveloped and cannot be maintained due to the loss of *ezh2*, followed by lethality around 10–12 dpf. We speculate that the *ezh2(sa1199)* stop mutation upstream of the WD-binding multimerization domain might cause a (more severe or) earlier phenotype, due to the function of the domain in PRC2 complex formation [17,18]. Monitoring the development of *ezh2(sa1199)* mutants, we concluded that there were secondary problems in the embryos, such as unexplained and non-specific edemas in wild types and mutants alike. The renewal of the line by selective breeding, out-crosses with wild types, and screening against the non-specific phenotype eliminated these edemas in wild type and *ezh2* mutant embryos alike. However, following several experiments, we did not observe any *ezh2* phenotype as seen and reported in published work on the (maternal) zygotic *ezh2(hu5670)* and zygotic *ezh2(ul2)* mutant alleles [16,36,37].

The *ezh2(sa1199)* line we first worked with showed multi-level problems regardless of genotype, and were most likely caused by background mutations after random ENU mutagenesis. The rate and frequency of mutations upon ENU treatment can be highly variable and dependent on the experimental conditions [51]. Edemas in zebrafish have been associated with environmental [52] or morpholino-induced toxicity [53], loss of gene function [54,55], lymphatic system and kidney failures [56,57], and they often coincide with tail curvature [58]. We speculate that the lethality and edemas observed at 2 dpf relate to background mutations.

The selectively bred *ezh2(sa1199)* line, interestingly, survived into adulthood with no apparent embryonic or larval phenotype. This observation contrasts with published research on the *ezh2(sa1199)* allele: *ezh2(sa1199)* mutants showed decreased Ezh2 protein and H3K27me1/2/3 levels at 2 dpf, and lethality at 7 dpf [44]. Moreover, the loss of *ezh2* affected the expression of circadian clock genes [44]. Our results do not corroborate the abovementioned study, and rather matches current observations made by the Wellcome Trust Sanger Institute, which indicate no phenotype until 5 dpf for this allele during the preparation of this manuscript ([9], https://www.sanger.ac.uk/sanger/Zebrafish_Zmpgene/ENSDARG00000010571#sa1199).

Although the *ezh2(sa1199)* mutants reached adulthood, they manifested an “open mouth” phenotype. Craniofacial abnormalities can be caused by PcG mutations, amongst others [47,48,49]. As these mutants were otherwise seemingly healthy adults, we concluded that the ‘open-mouth’ phenotype does not affect food intake.

When we detected abnormal water copper levels (resulting from a placement of copper pipes) in the zebrafish system, we evaluated copper as a possible cause for craniofacial abnormalities in the *ezh2(sa1199)* mutant adults. Effects of copper exposure on zebrafish embryos and adults have been studied by many research groups. In adult zebrafish, decreased oxidative capacity in liver and gill are observed upon water copper exposure [59]. Importantly, disruption of copper metabolism in early development as opposed to overexposure leads to pharyngeal arch deformities [60,61]. These findings illustrate the possibility that copper intoxication may have resulted in a jaw phenotype in this study. Further, the likelihood that *ezh2(sa1199)* mutants are more sensitive to environmental toxicity than wild types, which could be caused by lower H3K27me3 levels at 7 dpf, might explain the mutant-specific phenotype seen in this study.

Whole mount in-situ hybridization (WISH) for *ezh2* in 3 dpf embryos, and RT-qPCR on 70 dpf adults show that there is no reduction in *ezh2* mRNA levels in *ezh2(sa1199)* mutants. Western blot analysis on 70 dpf mutant adults further indicates that Ezh2 protein is translated. These two experiments were done in different *ezh2(sa1199)* lines from different age groups; the WISH experiment at 3 dpf in the line with non-specific edemas, the latter 70 dpf experiments in the line which survived until adulthood. We speculate similarities in the transcription and translation patterns between these two different generations of the *ezh2(sa1199)* line. Taken together, the results of the two experiments strongly suggest that the R18STOP mutation in the *ezh2(sa1199)* allele does not result in a loss-of-function mutation, as *ezh2* mRNA and Ezh2 protein were both present in these *ezh2* mutants.

There could be many reasons for a nonsense mutation to retain mRNA and protein expression. After transcription, NMD might be bypassed and a full-length protein might be translated, which could function just sub-optimally or normally [62]. In fact, many nonsense mutations are capable of bypassing NMD, and are tolerant to loss of function by presently unknown mechanisms [63–65]. Accumulating evidence suggests that nonsense mutations close to the N-terminus might be more likely to bypass NMD; in some cases, translation can be re-initiated at a downstream start codon [66,67]. In human cancer cells, NMD efficiency drops to 35% when the premature termination codon lies in the first 200 nucleotides, as opposed to 93% NMD efficiency for more downstream mutations [68]. The R18STOP mutation of the *ezh2(sa1199)* allele is located at Ezh2 N-terminus, which is highly conserved amongst vertebrates [16]. This conservation indicates high functionality and decreases the likelihood of exon skipping or alternative splicing. Indeed, there are no documented zebrafish *ezh2* transcript variants which skip the transcription of the first two exons (Ensembl Release 92, April 2018).

According to our findings, the nonsense mutation in the *ezh2(sa1199)* allele is not in concert with the presence of *ezh2* mRNA and Ezh2 protein in these mutant zebrafish. This study further emphasizes the importance of taking possible background and/or linked mutations into account in mutagenesis screens, and their correlation with (non-specific) phenotypes. Taken together, the presence of the Ezh2 mRNA and protein and the lack of a larval phenotype in mutants indicates that the *ezh2(sa1199)* allele is not a suitable model to study loss of *ezh2* function in zebrafish.

## Supporting information

S1 Fig Water quality measurements and Western blot analysis. **A.** Fold changes in element concentrations (ppb) in the water system which housed *ezh2(sa1199)* zebrafish. During regular water quality checks, Aluminum (Al), Calcium (Ca), Cadmium (Cd), Copper (Cu), Iron (Fe), Potassium (K), Magnesium (Mg), Manganese (Mn), Sodium (Na), Phosphorus (P), Sulfur (S), Silicium (Si), and Zinc (Zn) absorptions were measured and element concentrations were calculated. The graph shows the ratio of element concentrations between erroneous and correct piping. Erroneous piping in the water supply led to 33.46-fold higher than normal Copper concentrations. During this time, an open mouth phenotype was observed specifically in *ezh2(sa1199)* mutant adult zebrafish. **B.** Expression of Ezh2 protein in homozygous *ezh2(sa1199)* mutants. The presence of Ezh2 (top panel) and β-actin (bottom panel) was tested by Western blot analysis in wild type (*ezh2*^*+/+*^, N = 3, left) and mutant (*ezh2*^*sa1199/sa1199*^, N = 4, right) body, intestine, and head of single fish at 70 dpf. Ezh2 expression persisted only in the head of wild types and mutants. β-actin protein was used as a loading control. The figure shows the complete Western blot which was shown in part in Fig 3D upper panel, and includes additional intestinal samples which however were subject to autolysis, and head samples, which do not show Ezh2 expression. **C.** Western blot analysis in 7 dpf wild type (*ezh2*^*+/+*^) and mutant (*ezh2*^*sa1199/sa1199*^) larvae depicting the presence of the H3K27me3 mark (upper panel). In mutants, there is a 41.8% decrease in H3K27me3 levels, normalized against loading control β-actin (lower panel). The figure shows the complete version of the Western blot analysis depicted in Fig 3D lower panel (10 μl, right), with an additional dilution (5 μl, left). **D.** Western blot analysis of Ezh2 and β-actin in 24 hours-post fertilization (hpf) wild type (*ezh2*^*+/+*^, N = 2) and maternal-zygotic *ezh2* mutant (*MZezh2*^*-/-*^, N = 2) embryos [16,50], demonstrating that the antibody used in Fig 3D and S1B Fig is able to recognize the loss of the Ezh2 protein in established Ezh2 mutations, which are subject to NMD.
(EPS)

S1 Table List of RT-qPCR primers. Forward and reverse RT-qPCR primers for *ezh1*, *ezh2*, *ef1a*, *b-actin*, used for the analysis of relative *ezh1* and *ezh2* expression in 70 dpf wild type and *ezh2(sa1199)* mutant siblings.
(XLSX)
